# Spatially Varying and Scale-Dependent Relationships of Land Use Types with Stream Water Quality

**DOI:** 10.3390/ijerph17051673

**Published:** 2020-03-04

**Authors:** Se-Rin Park, Sang-Woo Lee

**Affiliations:** 1 Graduate Program, Department of Forestry and Landscape Architecture, Konkuk University, Gwangjin-Gu, Seoul 05029, Korea; 2 Department of Forestry and Landscape Architecture, Konkuk University, Gwangjin-Gu, Seoul 05029, Korea

**Keywords:** watershed land use, riparian area, water quality parameter, multi-scale analysis, geographically weighted regression

## Abstract

Understanding the complex relationships between land use and stream water quality is critical for water pollution control and watershed management. This study aimed to investigate the relationship between land use types and water quality indicators at multiple spatial scales, namely, the watershed and riparian scales, using the ordinary least squares (OLS) and geographically weighted regression (GWR) models. GWR extended traditional regression models, such as OLS to address the spatial variations among variables. Our results indicated that the water quality indicators were significantly affected by agricultural and forested areas at both scales. We found that extensive agricultural land use had negative effects on water quality indicators, whereas, forested areas had positive effects on these indicators. The results also indicated that the watershed scale is effective for management and regulation of watershed land use, as the predictive power of the models is much greater at the watershed scale. The maps of estimated local parameters and local R^2^ in GWR models showcased the spatially varying relationships and indicated that the effects of land use on water quality varied over space. The results of this study reinforced the importance of watershed management in the planning, restoration, and management of stream water quality. It is also suggested that planners and managers may need to adopt different strategies, considering watershed characteristics—such as topographic features and meteorological conditions—and the source of pollutants, in managing stream water quality.

## 1. Introduction

Stream water quality is influenced by complex interactions among various natural (e.g., weather, soil type, slope, and elevation) and anthropogenic factors (e.g., land use/cover types, changes and intensity in the watershed) [1,2]. Particularly, land use/land cover (LULC) change in the watersheds has been a focal area of study for decades among researchers, land planners, and stream managers because of its significant impacts on stream water quality. LULC changes in watersheds can alter watershed characteristics [3,4], thereby affecting various physical and biochemical stream characteristics, such as water temperature, nutrient/chemical concentration, sediment regime, stream geomorphology, aquatic habitat, and ecological biodiversity [3,5,6,7,8,9,10,11,12,13].

Different land use types determine the type and intensity of human activities, and the source of pollutants transported into streams, such as nutrients, sediments, chemicals, and pesticides. Impervious surfaces within a watershed are a key indicator of water quality. Impervious surfaces in urban areas prevent the natural runoff mechanisms from infiltrating water and lead to the flashiness of stream charge, increase the amount of discharge, shorten the run-off peak time, and lead to water quality degradation [14,15,16,17,18,19,20,21]. Agricultural land uses and raising livestock have also been shown to be a significant non-point source of sediments [16,18,19,20,21,22,23]. Forests, on the contrary, have various positive effects on streams, such as improving water quality [24,25], mitigating water quality degradation [18,26,27,28], and reducing sediment yield and pollutant loading in watersheds [27,29,30,31,32,33].

Land use impacts on streams vary in the spatial scale because streams are hierarchical systems surrounded by spatially heterogeneous landscape. Although multiple spatial scales, including sub-watersheds and riparian buffer zones, have been commonly applied in previous studies [3,11,34,35,36,37,38,39], there is still an ongoing debate regarding whether or not land use near streams has a greater influence on water quality than on the entire watershed. Although the buffer widths varied across studies, previous research has shown that land use within the riparian buffer zone has a greater influence on the water quality than on the entire watershed [3,16,17,18,40,41,42]. These effects on water quality are diverse between countries and the regions, water quality indicators, watershed characteristics, and watershed boundary scales [1,43,44]. Therefore, it is critical to understand the impacts of land use on water quality at multiple spatial scales to implement scale-appropriate strategies for water quality improvement and watershed management.

For investigating the effects of watershed and riparian characteristics on stream water quality, most previous studies adopted conventional statistical methods, such as the ordinary least squares regression (OLS) and Spearman’s rank correlation analysis [25,45,46,47]. OLS is one of the most commonly used statistical techniques for examining relationships between dependent and independent variables and identifying key factors in explaining the variance of stream water quality [47]. One critical assumption of OLS-based statistical methods is that the effects of land use on water quality are constant over space. However, the impacts of land use on water quality might vary over space because of the large number of factors and the complexity of the processes involved [48,49,50,51,52,53,54,55]. One possible way to handle these spatially varying effects of land use on water quality is through geographically weighted regression (GWR) [5,47,49,52,56,57]. In fact, numerous studies have reported that land use effects on stream water quality are complex and specific to the site, region, and landscape (e.g., References [20,58,59,60,61,62]). Nonetheless, GWR enables analysis of the spatially varying relationships between land use and stream water quality. It can overcome the deficits of conventional statistical methods (i.e., OLS-based methods) and be a powerful tool for providing space-specific critical knowledge for managing land use at various scales (e.g., site, region, landscape, and country). Despite its significance, the spatial variation of land use effects on water quality has not been extensively investigated [5,49]. 

Taking all of the above into account, the aim of this study is to investigate the relationships between land use types (i.e., urban, agricultural, and forested areas) and water quality indicators (i.e., BOD, DO, NH_3_-N and PO_4_-P) at two spatial scales (i.e., the watershed scale and riparian buffer scales) using two statistical methods (i.e., OLS-based models and GWR models). Sound watershed and stream management should be practiced based on an accurate assessment of the link between land use characteristics and stream water quality. This has become increasingly difficult because of many different land use practices in watersheds and their complex interactions [63,64,65]. In this regard, the results of this study can provide critical insights into preparing sustainable stream and watershed management guidelines for planners, managers, and decision makers.

## 2. Materials and Methods

### 2.1. Study Area 

South Korea is located between 35°74’ N and 127°46’ E, with an area of approximately 100,210 km^2^. As in any monsoon system, there are four seasons with distinctive seasonal characteristics in terms of temperature, precipitation, and wind speed. The annual average temperature ranges from 10 to 15 °C, and the average precipitation is between 1200 and 1800 mm. August is the hottest month of the year, with peak temperatures in the range of 23 to 26 °C, whereas, January is the coldest month with temperatures falling to the range of −6 to 3 °C. During summer (June–September), heavy rain, typhoons, and humid weather contribute approximately two thirds of the total annual rainfall. Winter can be characterized by dry weather and cold temperature, due to the northwest winds from Siberia. Thus, there are considerable fluctuations in precipitation and stream flows between seasons [66,67].

**Figure 1:**
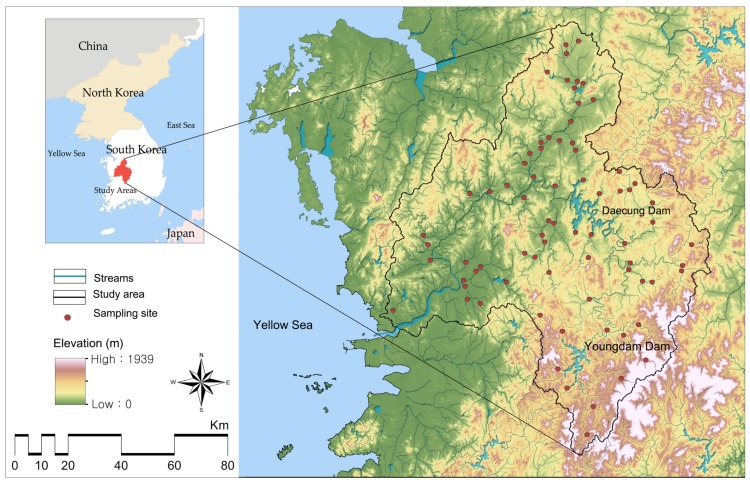
Geum River national watershed management region, topography, and sampling sites in the National Aquatic Ecological Monitoring Program in Korea.

There are four major rivers in South Korea: The Han, Nakdong, Geum, and Youngsan-Seomjin Rivers. The Geum River is the third biggest river in South Korea, covering the Midwest region of South Korea with a drainage area of 9915 km^2^ (Figure 1) and the Yellow Sea to the west of the watershed. The mainstream of the Geum River is 398 km long and has long tributary channels. More than 50% of the Geum River is occupied by forest areas, followed by agricultural areas. When compared with other major rivers, the Geum River basin is relatively flat with low altitudes, and most watersheds in the areas are occupied by agricultural lands. Hence, it is plausible that large amounts of synthetic fertilizers and manures are commonly released into streams from agricultural areas [68]. The most common type of water use in the Geum River basin is for agricultural water supplies, as the population in the basin was 6497 million as of 2014, accounting for about 12.4% of Korea’s population [69].

### 2.2. Spatial Scale of Analysis 

As shown in many previous studies, the relationships between land use and water quality are scale-dependent. Although multiple spatial scales, including watershed, riparian zones, and a series of buffers have been studied previously, the question of whether land use near streams has a greater influence on water quality than that in the entire watershed is still controversial (e.g., References [1,11,16,17,18,38,40,43,44,70]). For example, a number of studies argued that land use on the buffer scale was more important for stream water quality than that in the entire watershed because land use in the buffer zone has the strongest influence on streams, and pollutants, nutrients, and sediments loaded from watersheds could be filtered, infiltrated, or absorbed by vegetation and soils before they reached the stream [16,17,18,38,40,67,70]. However, buffer distance was never agreed upon among previous studies [16,17,18]. Some other studies reported that land use on the watershed scale explained the better water quality of the streams than land use on the buffer scale [1,11,44].

For monitoring purposes, the Korean Ministry of Environment (MOE) has identified and hierarchically structured watersheds across the entire country, including the national watershed management regions (NWMRs), based watershed management regions (BWMRs), and sub-watershed management areas (SWMAs). In addition, since 2007, the Korean MOE has designated areas within 500 m of buffers as riparian zones, to preserve stream water quality. For the current study, we decided to use SWMAs as the study unit, because they are the basic units of watershed management for local governments and the MOE. We computed the percentage of land use at two spatial scales, including the 500 m riparian buffer scale (e.g., References [17,67]) and sub-watershed scale (i.e., SWMAs; [49]). Thus, both the 500 m buffer scale and SWMAs are critical spatial units for stream water protection and watershed management in Korea.

### 2.3. Water Quality Parameters 

The MOE in Korea has monitored various indicators, including plants, geomorphological characteristics (e.g., velocity, width, and depth), biochemical characteristics (e.g., BOD, COD, DO, NH_3_-N, NO_3_-N, PO_4_-P, TN, TP, pH, SS, etc.), and biological indicators (e.g., benthic diatoms, macroinvertebrates, and fish) throughout the country under the National Aquatic Ecological Monitoring Program (NAEMP) since 2007 (for more detailed information on NAEMP, see Lee et al., Reference [71]). Under the NAEMP, sampling sites were selected based on the size of river systems within the watersheds, the land uses, and specific stream management interests.

We used 2014 monitoring data for matching with the most up to date LULC data released by the MOE. Sampling in the Geum River basin was conducted by multiple universities for two weeks (April 28–May 9) in spring under NAEMP and MOE. Out of 170 sampling sites in the Geum River basin, we selected 76 sampling sites of tributary streams for analysis, omitting the sampling sites on the main river because of substantially different stream environments and sizes [67]. Four water quality parameters (i.e., BOD, DO, NH_3_-N, and PO_4_-P) were used as independent variables, and these water quality parameters have been used most commonly in many previous studies for representing stream water quality (e.g., References [25,61,72,73,74,75,76,77]).

### 2.4. Computing the Percentage of Land Use Types

To compute the proportions of land use types in watersheds, we integrated the digital LULC map released by the MOE. The LULC map was generated using the Landsat Thematic Mapper (30 m resolution) and Indian Remote Sensing (IRS) – 1C pan-chromatic (5.8 m resolution) images, taken in 2007 and updated in 2015 and 2016 [67]. According to the MOE classification, land use types were divided into seven categories and 23 sub-categories. For the current study, we used three main categories: (a) Urban areas (i.e., industrial, residential, and commercial areas), (b) agricultural areas (i.e., farms and rice paddy), (c) forested areas (i.e., deciduous, coniferous, and mixed forests). The percentage of each land use within each SWMA and riparian buffer area was computed in ArcMap and converted into proportional data for analysis.

### 2.5. Analysis and Model Estimation 

Prior to estimating the models, the Pearson correlation analysis was used to explore the simple relationships between the percentages of land use types and water quality parameters. GWR modified the traditional regression model, such as OLS to address the spatial variations among variables and display local rather than global statistics [56,57]. To estimate the OLS-based regression models, the computed percentages of land use types at SWMA and buffer scales were regressed to each water quality parameter (i.e., BOD, DO, NH_3_-N, and PO_4_-P) with the step-wise option using the SPSS 25 software (IBM Corp., Armonk, NY, USA). GWR models for each water quality parameter were estimated using the embedded software ArcToolbox in ArcMap. All mappings and spatial analyses were also conducted using ArcMap 10.6.1 (ESRI Inc., Redlands, CA, USA). In addition, the spatial variations of the parameters and the local coefficient of determination (R^2^) of the estimated GWR models were visualized in ArcMap 10.6.1. In general, several hundred datasets should be considered to apply GWR to obtain best results. However, numerous previous studies have applied GWR with smaller datasets, which is insufficient for GWR analysis [55,78]. Because the dataset in this study comprises stream monitoring data, the number of sampling sites is limited and should be located along the rivers. We found that it is difficult to apply spatial bootstrapping methods for this case. Alternatively, we bootstrapped the coefficients of estimated GWR models and computed confidence intervals to reduce the uncertainty, due to the small-sized dataset. Bootstrapping was carried out using the boot package in R for 3000 resamples.

In order to compare the performance of the estimated OLS and GWR models, we selected three criteria: R^2^ values, Akaike’s Information Criterion (AICc), and spatial autocorrelation of residuals (Moran’s *I*). Greater R^2^ and lower AICc values indicate that the estimated model closely represents the actual nature of the relationships between land use types and water quality [57,79]. Moran’s *I* ranges from −1 to 1, and a value close to −1 or 1 indicates that residuals are spatially dependent [57]. In contrast, for the residuals of estimated models, a value of Moran’s *I* close to zero suggests that the residuals are spatially independent.

## 3. Results

### 3.1. Descriptive Statistics and Spatial Distributions 

**Figure 2:**
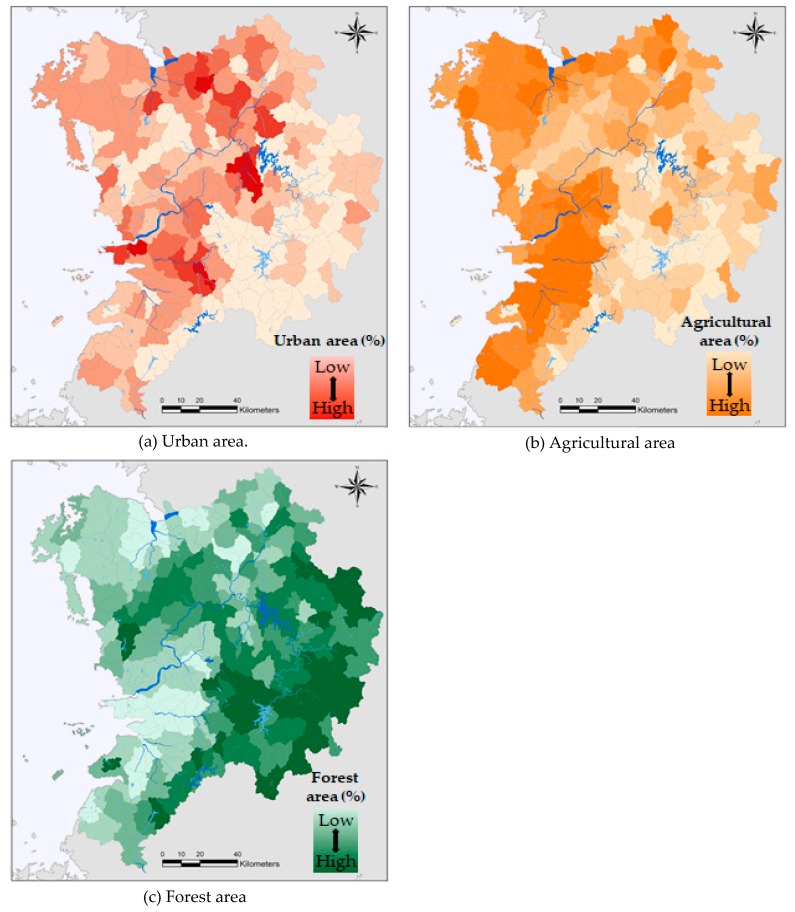
Spatial distribution of urban, agricultural, and forest areas (%) in the study area.

Water quality, including biochemical oxygen demand (BOD), dissolved oxygen (DO), ammoniacal nitrogen (NH_3_-N), and phosphate (PO_4_-P), varied significantly among sampling sites (Table 1). The mean values of BOD, DO, NH_3_-N, and PO_4_-P within the study area were 2.61, 9.74, 0.13, and 0.03 mg/L, respectively. The result indicated that the water quality is good based on the BOD and DO values measured using organic matter, but poor based on the NH_3_-N and PO_4_-P values measured using nutrients. The relative proportions of each type of land use at two different scales varied across the study areas. The mean values of the urban, agricultural, and forested areas at the SWMA scale were 7.73%, 29.47%, and 56.03%, respectively; i.e., the dominant land use type was a forest. The standard deviations of agricultural and forested areas were high, suggesting that there is a greater variance across the watershed. The mean values of the urban, agricultural, and forested areas at the riparian scale were 10.09%, 43.62%, and 46.29%, respectively. Compared with land use at the SWMA scale, the proportion of urban and agricultural areas was higher, and that of forested areas was lower. Figure 2 shows the spatial distributions of the land use, including urban, agricultural, and forested areas. Urban areas were mainly observed in the center and at the northern and western sides of the study area, which has a large population. The spatial distribution of agricultural areas was high in the western region of the study area, with mild slopes and low elevations. The spatial distribution of forested areas was observed in steep slopes and high elevations and revealed a gradual increase from west (low) to east (high).

### 3.2. Correlations between Land Use Types and Water Quality Parameters

The percentage of urban and agricultural areas was positively correlated with the concentrations of BOD, NH_3_-N, and PO_4_-P, whereas, the percentage of forested areas showed opposite relationships with the concentrations of BOD, NH_3_-N, and PO_4_-P at the SWMA scale. In addition, the correlation between the concentration of DO and the percentages of agricultural and forested areas was negative and positive, respectively. Therefore, the water quality status was likely poor if the percentage of developed areas, including urban and agricultural areas in watersheds was high. On the contrary, the water quality was likely better when watersheds were dominated by more forests. However, the percentage of urban land uses in watersheds showed relatively weak relationships with the concentration of PO_4_-P. No significant relationship between the percentage of urban land use and the concentration of DO was observed at the watershed scale. Further, it was possible to observe a similar pattern of the relationship at the riparian scale. Specifically, the percentage of urban areas was positively correlated with the concentration of BOD at the riparian scale, whereas, the percentage of urban land uses at the riparian scale showed no significant relationship with the concentrations of DO, NH_3_-N, and PO_4_-P. 

The percentage of agricultural areas at the riparian scale was positively associated with the concentrations of BOD, NH_3_-N, and PO_4_-P, and the negatively correlated with the concentration of DO. We also observed negative relationships between the percentage of forests in riparian areas with the concentrations of BOD, NH_3_-N, and PO_4_-P, as well as positive associations with DO. Therefore, stream water quality was likely better if riparian areas were covered by less urban and agricultural areas, but more by forests. These results suggested the positive effects of forests and the negative effects of urban and agricultural areas on both the watershed and riparian scales. It was noteworthy that urban areas showed relatively weaker relationships with water quality parameters than agricultural and forest areas on both scales. In addition, the percentage of land use types showed stronger relationships with water quality parameters on the watershed scale than on the riparian scale (Table 2).

### 3.3. Estimated OLS Models at Two Spatial Scales

On both scales, the main land use types affecting stream water quality were agricultural or forest areas, whereas, urban land use did not appear to be the primary land use type influencing stream water quality (Table 3). Specifically, the percentage of forested areas significantly lowered the concentration of BOD of streams at the watershed scale in the study areas (b = −0.057, *β* = −0.638, *p* < 0.01). Approximately 40% of the variance of the BOD concentration of streams was explained by the percentage of forested areas in the watershed, and the percentage of urban and agricultural areas did not appear to be significant variables in the estimated BOD regression model. The positive effect of the forested areas was also observed in the NH_3_-N model at a watershed scale. In the estimated model for NH_3_-N, the percentage of forested areas in the watershed considerably reduced the concentration of NH_3_-N in streams (b = −0.006, *β* = −0.508, *p* < 0.01), and the coefficient of determination of the model was 0.25. Meanwhile, the concentration of DO was significantly decreased by the percentage of agricultural areas in the watershed (b = −0.045, *β* = −0.503, *p* < 0.01). On the contrary, the percentage of agricultural areas in the watershed (b = 0.001, *β* = 0.666, *p* < 0.01) increased the concentration of PO_4_-P in streams. The coefficients of determination of DO and PO_4_-P models were 0.25 and 0.44, respectively.

At the riparian scale, the percentage of forested areas appeared to lower the concentration of BOD in the estimated BOD regression model (b = −0.044, *β* = −0.564, *p* < 0.01) and explained approximately 31% of the variance of the BOD concentration in streams. In the estimated DO, NH_3_-N, and PO_4_-P models at the riparian scale, the percentage of agricultural areas appeared to be the main determinant of the concentrations of DO, NH_3_-N, and PO_4_-P. Specifically, the percentage of agricultural areas significantly reduced the concentration of DO (b = −0.041, *β* = −0.572, *p* < 0.01) and increased the concentrations of NH_3_-N (b = 0.005, *β* = 0.461, *p* < 0.01) and PO_4_-P (b = 0.001, *β* = 0.637, *p* < 0.01) in streams. The coefficients of determination of the estimated regression models for DO, NH_3_-N, and PO_4_-P were 0.32, 0.2 and 0.4, respectively. 

Overall, the estimated OLS models for BOD, DO, NH_3_-N, and PO_4_-P indicated that the percentages of agricultural and forested areas at both scales were primary land use types in determining the water quality of the stream. These estimated regression models clearly showed the positive effects of forested areas and negative effects of agricultural areas on water quality at the watershed and riparian scales. In terms of the coefficient of determination, land use types at the watershed scale explained the stream water quality better than those at the riparian scale, except for the concentration of DO. 

### 3.4. Estimated GWR Models at Two Spatial Scales

The estimated GWR models for BOD, DO, NH_3_-N, and PO_4_-P at the watershed and riparian scales suggested that the effects of forested and agricultural areas on stream water quality varied significantly over the study areas (Table 4). In particular, the coefficient of the percentage of forested areas for BOD at the watershed scale varied from −0.075 to 0.009 over space, suggesting that forested areas might increase the concentration of BOD in streams under certain circumstances (R^2^ = 0.4). A similar variance was observed for the coefficient of the percentage of forested areas at the riparian scale, but the range of the variance (−0.048 to −0.002) was relatively small (R^2^ = 0.31). The estimated GWR model for DO indicated a negative effect of the percentage of agricultural areas in the watershed (R^2^ = 0.24). The negative effect of agricultural areas on DO varied considerably, from −0.079 to 0.001 across the study areas, suggesting that the percentage of agricultural areas in the watershed might increase the concentration of DO under certain circumstances. However, the coefficient of agricultural areas for DO in riparian areas varied within a relatively small range (−0.065 to −0.017; R^2^ = 0.32). The coefficient of the percentage of forested areas for NH_3_-N at the watershed scale significantly varied from −0.007 to −0.005 over space, suggesting that forested areas might reduce the concentration of NH_3_-N in streams (R^2^ = 0.25). Moreover, the estimated GWR model for NH_3_-N showed a negative effect of the percentage of agricultural areas at riparian scales (R^2^ = 0.20), indicating that the negative effect of agricultural areas on NH_3_-N varied over space. The percentage of agricultural areas at both the watershed and riparian scales appeared to increase the concentration of PO_4_-P in the estimated GWR models. A similar variance of the coefficient of agricultural areas was observed at both the watershed (R^2^ = 0.44) and riparian scales (R^2^ = 0.40).

Overall, we observed that the effects of land use types in the watershed and riparian areas on water quality varied over space. Furthermore, we observed that the GWR models at the watershed scale performed better than those at the riparian scale in terms of R^2^ and AICc values, except for the DO models (Table 4). All model performance indicators of GWR models for BOD and PO_4_-P consistently indicated better performance of the watershed scale model over the riparian scale model. On the contrary, the GWR models for DO suggested that riparian scale model performed better than the watershed scale model. However, model performance indicators of the GWR models for NH_3_-N were inconsistent between the watershed and riparian scales. It was observed that GWR model at the watershed scale performed better than that at the riparian scale in terms of R^2^ and AICc values. However, the GWR models at the riparian scale performed better when considering Moran’s *I* values.

We bootstrapped the coefficients of estimated GWR models and computed confidence intervals to reduce the uncertainty, due to the small-sized dataset (Table 5). The results of the bootstrap analysis indicated that the estimated coefficients of land use percentage in GWR models for BOD, DO, NH_3_-N, and PO_4_- P at both scales fall within the confidence interval range (2.5%–97.5%).

### 3.5. Comparison between OLS and GWR Models

At the watershed scale, all model performance criteria indicated better performance of the GWR model over the OLS model for BOD. Specifically, the coefficient of determination of the OLS model (R^2^ = 0.4) for BOD was improved in the GWR model (R^2^ = 0.5). AICc and Moran’ *I* values (261.66 and 0.21, respectively) were also decreased in the GWR model (AICc = 254.44, Moran’s *I* = 0.004), indicating considerable improvement of the model performance in delineating the relationship between land use types and the concentration of BOD. We observed similar improvement of the estimated models between the OLS and GWR models for DO at the watershed scale. The R^2^ values of the OLS and GWR models for DO at the watershed scale were 0.24 and 0.44, respectively, indicating that the GWR model performed better in terms of explaining the variance of the concentration of DO than the OLS model. The lower AICc and Moran’s *I* value of the GWR model compared with those of the OLS model for DO confirmed the better performance of the GWR model. However, a significant difference was not found between GWR and OLS models for NH_3_-N at the watershed scale, even though all criteria of the two models for NH_3_-N at the watershed scale were almost identical. In addition, the higher R^2^ and the lower AICc of the GWR model for PO_4_-P compared with those of the OLS model suggested better performance of the GWR model in terms of explaining the relationship between land use type and concentration of PO_4_-P at the watershed scale. However, the Moran’s *I* value of the GWR model for PO_4_-P was improved in the OLS model at the watershed scale. These mixed results of model performance criteria suggested no considerable difference between the two models in explaining the relationship between the concentrations of PO_4_-P with land use type in streams at the watershed scale. Hence, we observed considerable improvement of the GWR over the OLS model for BOD and DO at the watershed scale. However, there was no significant difference between the models in terms of explaining the relationships of the concentrations of NH_3_-N and PO_4_-P and land use type at the watershed scale.

At the riparian scale, we observed very similar results indicating the better performance of the GWR than the OLS model for BOD and DO. The higher R^2^ value and the lower AICc and Moran’s *I* value of the GWR model for BOD and DO indicated its better performance relative to the OLS model in terms of explaining the variances of the concentrations of BOD and DO. However, we were not able to observe considerable differences between the GWR and OLS models in terms of explaining the variances of the concentration of NH_3_-N and PO_4_-P at the riparian scale. All model performance criteria of the GWR and OLS models for NH_3_-N and PO_4_-P were almost identical (Table 6).

Overall, the GWR models for BOD and DO performed considerably better than the OLS models, at both the watershed and riparian scales. However, no considerable difference between the two models in terms of explaining the concentrations of NH_3_-N and PO_4_-P was observed. Despite these mixed results, it could not be conclusively determined that land use effects on the concentrations of NH_3_-N and PO_4_-P in streams are constant over space.

## 4. Discussions 

### 4.1. Land Use Types and Water Quality

Based on the result of the OLS models employed in this study, it was found that agricultural and forested areas were the dominant factors influencing water quality variations at both scales considered, whereas, urban areas were not a good predictor. Several other studies have also noted the importance of the impacts of agricultural and forested areas in watersheds on water quality. The OLS models employed in this study for BOD and NH_3_-N at the watershed scale confirmed the results of previous research reporting positive effects of forested areas on water quality in watersheds. Previous studies have shown that greater proportions of forest coverage are associated with the health of aquatic ecosystems [48,80,81,82], and improvements in water quality [24,25], as forest coverage plays an important role in mitigating water quality degradation [18], and produces less sediment and pollutants [29]. Singh and Mishra [33] found that a decrease in the forest cover increased the quantity of sediment yield, nutrients, and chemicals affected by turbidity and total suspended solids. Specifically, the forest cover reduces nitrates, and phosphorous loading into the stream and increases DO concentration in the stream [27,83].

Our OLS models for DO and PO_4_-P at the watershed scale suggested that greater proportions of agricultural areas are associated with poorer water quality parameters, which is consistent with the results of previous publications. As agricultural nutrients from fertilizers and pesticides decrease DO, which can lead to the degradation of aquatic ecosystem habitats, it is critical for the concentration of DO to be appropriate in streams for the functioning of aquatic ecosystems. It was also reported in previous studies that agricultural areas have a strong positive influence on nitrogen and sediment loads from fertilizers, pesticides, herbicides, and diary manures in the cropland [84,85,86,87,88]. Specifically, animal wastes and domestic sewage from agricultural areas could be the primary sources of phosphorus, which greatly contributes to water quality degradation [20,83].

Many previous studies have shown that greater proportions of urban areas in watersheds are a key factor affecting water quality [15,89,90,91,92,93]. Previous results have shown that urban areas are closely related to water quality pollution indicators, such as nitrogen, phosphorus, and ammonia [41,44,45]. Additionally, DO concentration decreases because of the discharge of organic matter in urban areas [92]. Urban runoff contains a large number of pollutants accumulating on impervious surfaces, such as parking lots, roads, and housing, thus influencing water quality [94,95,96]. However, in the current study, there were no significant relationships between urban areas and water quality parameters in our OLS models.

Many studies have shown that urban and agricultural areas were found to be major causes of water quality degradation [15,87,92,94]. However, uncertainties regarding whether the urban or agricultural land use is more important for the water quality of the streams still remain [50,73]. Lee et al. [25] and Ding et al. [73] identified that there was a strong relationship between urban land use and water quality indicators, rather than agricultural land use, because of the significant contribution of rapid urbanization and farming management practices in the study area. In contrast, several other studies have reported that agricultural land use was a major consideration in water quality degradation [20,97,98]. In specific, Wan et al. [20] found that agriculturally developed areas lead to more pollutants than urban areas because of the lack of facilities in agricultural areas for the treatment of pollutants from domestic sewage and human and animal excreta. These mixed results are partly owing to the distinctive characteristics of each watershed. Furthermore, a few studies pointed out that the percentage of urban land use in watersheds could determine the intensity of land use impact [39,50,98], although this is still controversial. Osborne and Wiley [39] have reported that only 5% of urban areas in watersheds could explain most pollutant loadings. Hooda et al. [99] found that agricultural land use influenced water quality more than any other land use type, when the percentage of urban land use is less than 5%. In the current study, agricultural land use impacts on water quality parameters were observed to be more intensive than those of urban land cover at both the scales, because the urban land cover is low (about 7%) and agriculturally developed areas are extensive.

### 4.2. Scale Effect on Relationships between Water Quality and Land Use

As the watershed boundary is a critical factor for regulating and managing land use, and riparian zones are important as an aquatic-terrestrial ecotone, it follows that both the watershed and riparian scales should be considered [100,101]. Particularly, riparian forests have positive effects on stream water quality and stream health, such as reducing pollutant loading from various land use types, lowering stream water temperature, stabilizing stream banks, and providing physical habitats [28,83]. Riparian vegetation also reduces phosphorus, nitrates, and sediment loading into the stream [27,32,41]. However, riparian forests have been fragmented and are in danger of disappearing owing to human activities and land development; such a development can affect the characteristics of hydrological runoff processes by increasing flow velocity and decreasing residence time within riparian buffers [67]. The land use effect on water quality at the riparian scale, as identified in this study, verifies the findings of previous studies indicating the positive effect of riparian forests and the negative effect of agricultural areas in riparian zones.

In our study area, the proportion of each land use type at the watershed scale appears to be more important in determining water quality parameters than at the riparian buffer scale. We found that the predictive power of the models was much higher at the watershed scale, except for the DO parameter. Land use types have a stronger relationship with water quality parameters at the entire watershed than at the riparian buffer, which reflects the scale effects and the existence of the effective spatial scale. Despite many studies that have highlighted the importance of a multiscale analysis on the relationship between land use and water quality, uncertainties regarding whether the watershed or riparian buffer scale is more important in influencing water quality still exist [41,95,102]. Some studies have shown that land use in a riparian zone is a better predictor of water quality than in the watershed [17,103,104], although the effective buffer width is still a subject of significant debate. Other studies have emphasized that stream water quality is better explained by land use at the entire watershed than at riparian zone [25,40,95,105], as investigated in this study. Zhou et al. [94] also found that the effective spatial scale varied for the given water quality parameters. These numerous studies suggest that a multi-scale perspective must be adopted when establishing and implementing watershed management planning. Based on our study, the entire watershed management process is extremely necessary, aiming at water quality improvement, although it is still critical to prioritize the riparian zone for maintaining water quality.

### 4.3. Comparison between OLS and GWR Models

In this study, both the estimated OLS and GWR models (Table 6) strongly indicated that the agricultural and forested land use significantly impacted stream water quality. The GWR models for BOD and DO performed considerably better than the OLS models, but there was no considerable difference between GWR and OLS models in explaining the concentration of NH_3_-N and PO_4_-P. Compared with the OLS models for BOD and DO, the GWR models performed considerably better in explaining the spatial variance in the effect of land use on water quality. As previous studies have reported, the relationship between land use and water quality was not constant over space, but varied among sites depending on watershed characteristics [16,52,78,106,107]. Tu [55] also reported similar results, indicating that land use effects on water quality parameters could vary by location with different levels of urbanization. However, it is difficult to find the cause of the non-stationary effect, due to the complex interactions among numerous variables, such as topographic features, meteorological conditions, and the source of pollutants [108,109,110]. In other words, all watersheds and streams are affected by adjacent environments in different ways and to different degrees. Although there were no significant differences in the explanatory power and spatial autocorrelation between the OLS and GWR models for NH_3_-N and PO_4_-P in this study, the results of GWR models for NH_3_-N and PO_4_-P are still meaningful and can capture spatial variability among study areas and help explore the relationship between land use and water quality parameters. The spatial variation in the impacts of land use on water quality can explain factors that the OLS models are unable to identify. The results of GWR models can be adopted for environmental policymakers in terms of maintaining, controlling, and improving water quality.

### 4.4. Spatially Varying Relationships between Water Quality and Land Use 

**Figure 3:**
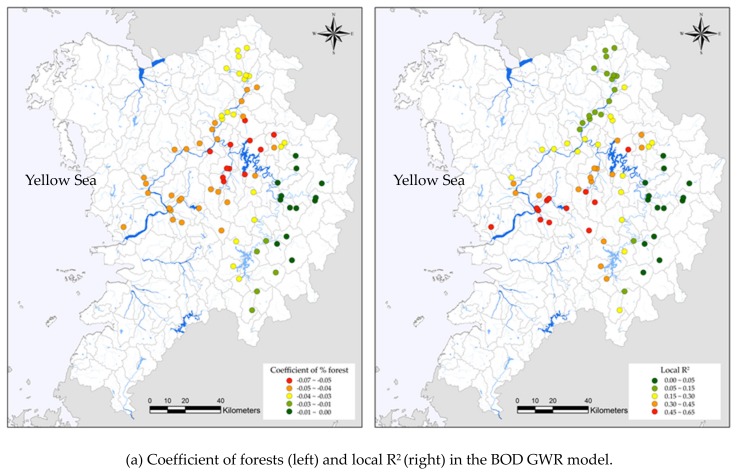

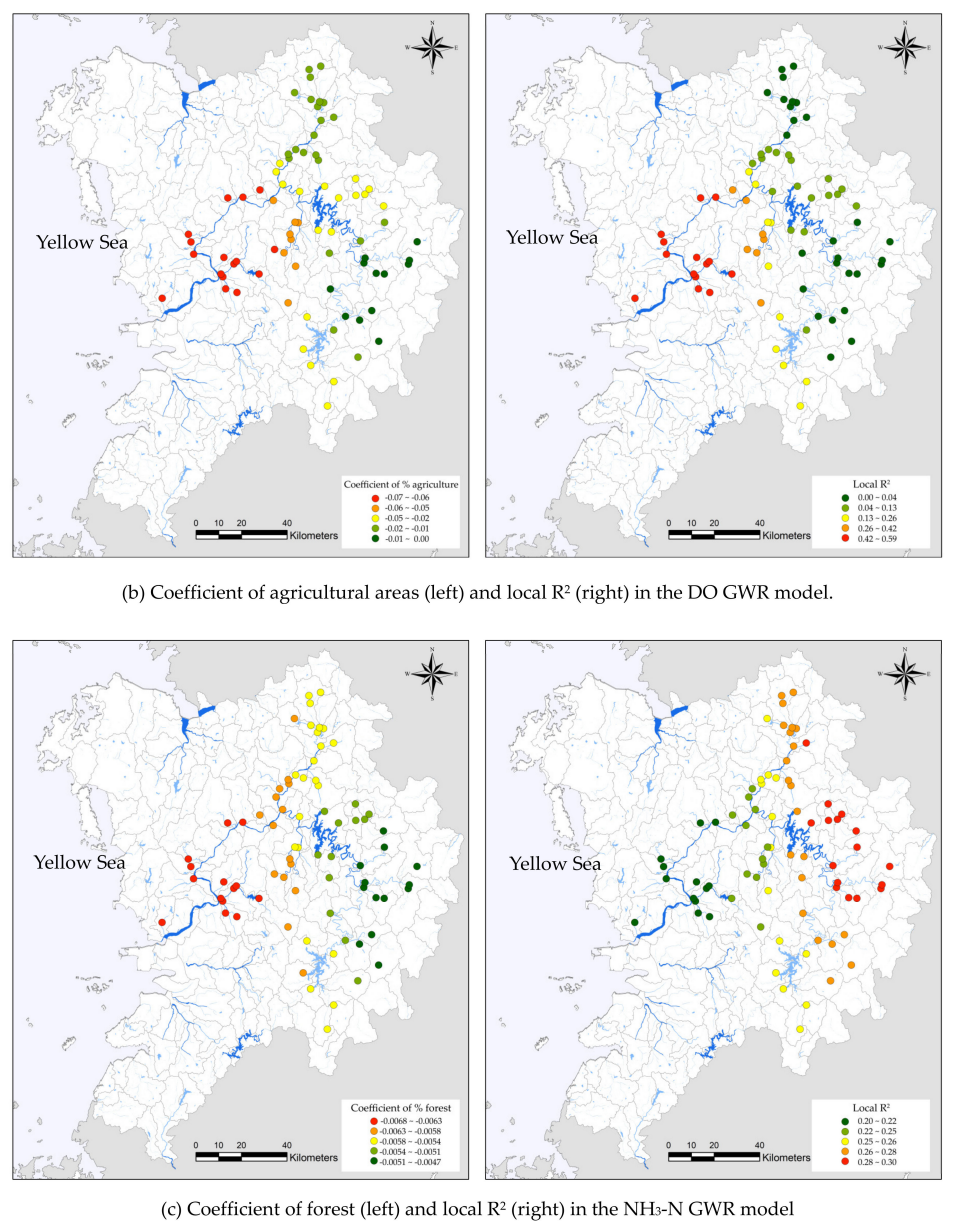

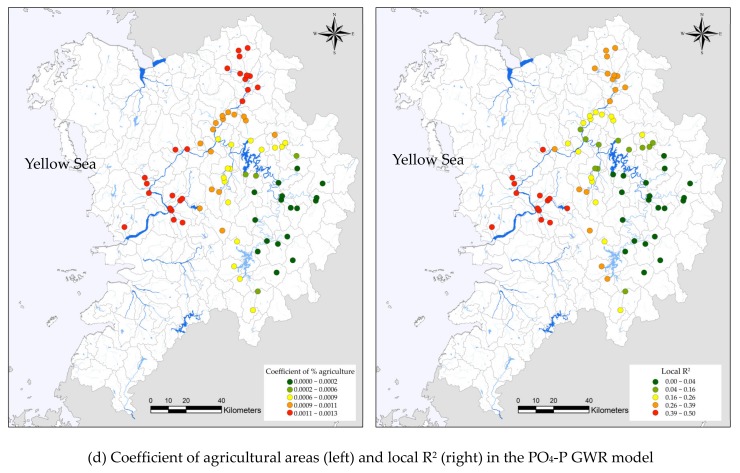
Spatial distribution of land use coefficient and local R^2^ in the estimated GWR models for BOD (**a**), DO (**b**), NH_3_-N (**c**), and PO_4_-P (**d**). The GWR model showed significant spatial variation of the land use coefficient and local R^2^.

In order to find the spatially varying relationships between land use and water quality, we generated maps of local parameter estimates and local R^2^ in GWR models for BOD, DO, NH_3_-N, and PO_4_-P (Figure 3). Maps of the local parameter estimates show the spatial variance of coefficients which represent the magnitude and direction of the relationship between land use and water quality. The local R^2^ value indicates the abilities of independent variables to explain the spatial variance in the water quality indicator at different sampling sites. In our study, spatial non-stationarity was present in the relationships between forested areas and BOD (Figure 3a). Forested areas had a strongly negative relationship with BOD over the study area. The red points indicated the stronger effects of forested areas on BOD. Higher coefficient values of the GWR model for the BOD were observed in less forested areas, while lower coefficient values were mainly located in highly forested areas. This indicated that forested areas were more important predictors of BOD in less forested watersheds than in highly forested watersheds. Higher R^2^ values (red dots) of the GWR model for BOD (Figure 3a) were observed mostly in the middle of the study area, while lower R^2^ values (dark green dots) were mainly located in the east part of the study area. The result indicated that the ability of forested areas to explain the spatial variation of the BOD greatly varied across the study area. There was also a clear spatial non-stationarity in the relationships between agricultural areas and DO (Figure 3b). Higher agricultural coefficient values were concentrated mostly in the middle of the study area, suggesting the higher effect of agricultural areas on DO. Higher R^2^ values in estimated GWR models for DO were observed in the middle areas.

The percentage of forested land exhibited negative relationships with NH_3_-N across the entire study area and varying over space (Figure 3c). The R^2^ values from the GWR model for the NH_3_-N showed clear spatial differences (Figure 3c). In the NH_3_-N model, higher R^2^ values were observed in less forested areas, whereas, lower R^2^ values were mainly located in highly forested areas. In addition, the percentage of agricultural areas showed significant positive relationships with PO_4_-P. In the PO_4_-P model, higher coefficient values were mainly observed in higher agricultural areas and lesser forested areas. The ability of agricultural land to explain the spatial variation in PO_4_-P varied significantly across the study area. Higher R^2^ values were in higher agricultural areas and in lesser forested areas.

**Figure 4:**
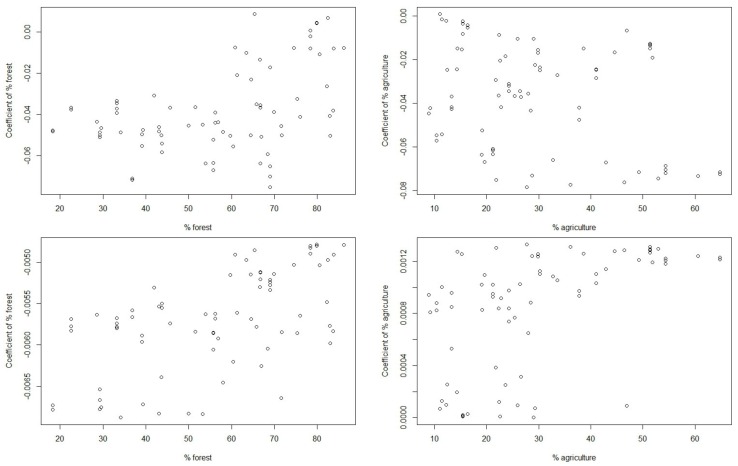
Scatterplots of the percentage of land use and the coefficients of land use in the estimated GWR models for BOD (upper left), DO (upper right), NH_3_-N (bottom left), and PO_4_-P (bottom right).

We generated scatterplots in order to showcase the relationship between the coefficients of land use in the estimated GWR models and the percentage of land use (Figure 4). Overall, we observed that the relationship was not linear. A *t*-test was used to compare the means of the percentage of land use of two groups: The group with percentages below the mean and the group with percentages above the mean (Table 7). We divided the percentage of land use into two groups divided by mean, and the difference in mean coefficients between the two groups were examined using the *t*-test. The result of the *t*-test indicated that the mean coefficients were significantly different, suggesting that the effect of land use on water quality would differ with the percentage of land use (except for the DO model). Specifically, the positive effect of forests on water quality is greater if forested areas in watersheds are greater than a certain percentage (i.e., the mean value in our cases). In other words, foresting in watersheds to improve water quality might not be effective if the forested areas are not large enough. However, it is noteworthy that the critical percentage of foresting in the watershed for improving water quality would depend on the various characteristics of the region, such as elevation, slope, soil types, and configuration of landscape elements.

## 5. Conclusions 

In this study, we examined the relationships between land use types and water quality indicators at two spatial scales, namely, the watershed and riparian scales, using two statistical methods. The results demonstrated that stream water quality is significantly related to agricultural and forested areas at both scales. The results of this study indicated that a higher degree of agricultural area negatively affects water quality, whereas, a higher percentage of forested areas has positive effects on stream water quality. The results also suggested that the effects of land use on water quality are scale dependent. Our study suggests that the entire watershed scale (SWMA scale) is a more effective spatial scale for assessing and predicting the impacts of land use types on water quality, when compared with the riparian buffer scale. As discussed in previous studies, it is critical to adopt a multi-scale perspective when establishing watershed management planning. Numerous previous studies reported that GWR models have better predictive performance than OLS models. Our study suggests that the GWR models for BOD and DO performed considerably better than OLS models, but there was no considerable difference in terms of explaining the concentration of NH_3_-N and PO_4_-P. However, maps of the local parameter estimate and the R^2^ values from GWR models provide a visualization of the spatially varying relationships among variables. The results of GWR models can serve as a useful tool for environmental policymakers in terms of maintaining, controlling, and improving water quality. Although sound watershed and stream management should be practiced based on the accurate assessment of the linkage between land use characteristics and stream water quality, this has been increasingly difficult because of many different land use practices in watersheds and their complex interactions. In this regard, the results of this study can provide critical insights into preparing sustainable stream and watershed management processes for planners, managers, and decision makers.

## Figures and Tables

**Table 1:** Descriptive statistics of measured water quality parameters and percentage of land use types at two different scales. SWMA, sub-watershed management areas.

Classification	Variables	Mean	S.D.	Min.	Max.
Water Quality Parameter	BOD (mgL^−1^)	2.61	1.69	0.30	7.70
	DO (mgL^−1^)	9.74	1.35	6.30	12.61
	NH_3_-N (mgL^−1^)	0.13	0.22	0.007	1.42
	PO_4_-P (mgL^−1^)	0.03	0.03	0.002	0.12
Land Use (SWMA scale)	Urban area (%)	7.73	6.80	0.65	30.05
	Agricultural area (%)	29.47	15.01	8.91	64.79
	Forested area (%)	56.03	19.07	18.31	86.19
Land Use (Riparian scale)	Urban area (%)	10.09	9.0	0.00	49.20
	Agricultural area (%)	43.62	18.81	12.12	86.04
	Forested area (%)	46.29	21.66	5.81	84.84

n = 76. S.D. = Standard Deviation, Min. = Minimum, Max. = Maximum.

**Table 2:** Pearson correlations between land use types and water quality parameters at SWMA and riparian buffer scales.

Scales	Land Use (%)	BOD (mgL^−1^)	DO (mgL^−1^)	NH_3_-N (mgL^−1^)	PO_4_-P (mgL^−1^)
SWMA scale	Urban	0.53 **	−0.14	0.33 **	0.29 *
	Agriculture	0.51 **	−0.50 **	0.49 **	0.67 **
	Forest	−0.64 **	0.40 **	−0.51 **	−0.63 **
Riparian scale	Urban	0.33 **	0.04	0.09	0.08
	Agriculture	0.49 **	−0.57 **	0.46 **	0.64 **
	Forest	−0.56 **	0.48 **	−0.44 **	−0.56 **

n = 76. * *p* < 0.05, ** *p* < 0.01.

**Table 3:** The estimated ordinary least squares (OLS)-based regression models at SWMA and riparian scales.

Water Quality	Estimated Regression	F-Value	*Adj*. R^2^
**SWMA scale**			
BOD (mgL^−1^)	−0.057 × %Forest ** + 5.79	50.91 **	0.40
DO (mgL^−1^)	−0.045 × %Agriculture ** + 11.07	25.03 **	0.24
NH_3_-N (mgL^−1^)	−0.006 × %Forest ** + 0.46	25.67 **	0.25
PO_4_-P(mgL^−1^)	0.001 × %Agriculture ** − 0.007	58.92 **	0.44
**Riparian scale**			
BOD (mgL^−1^)	−0.044 × %Forest ** + 4.657	34.47 **	0.31
DO (mgL^−1^)	−0.041 × %Agriculture ** + 11.534	35.91 **	0.32
NH_3_-N (mgL^−1^)	0.005 × %Agriculture ** − 0.1	20.01 **	0.20
PO_4_-P (mgL^−1^)	0.001 × %Agriculture ** − 0.012	50.55 **	0.40

** *p* < 0.01.

**Table 4:** Estimated GWR models for BOD, DO, NH_3_-N, and PO_4_-P at SWMA and riparian scales. The higher value of R^2^ and lower values of AICc and Moran’s *I* (absolute value) indicate better performance of the estimated models.

Indicator	BOD		DO		NH_3_-N		PO_4_-P	
	SWMA	Riparian	SWMA	Riparian	SWMA	Riparian	SWMA	Riparian
Mean C. ^a^	−0.039F	−0.029F	−0.037A	−0.035A	−0.006F	0.005A	0.0008A	0.0007A
Min. C. ^b^	−0.075F	−0.048F	−0.079A	−0.065A	−0.007F	0.004A	0.000A	0.0002A
Max. C. ^c^	0.009F	−0.002F	0.001A	−0.017A	−0.005F	0.006A	0.0013A	0.0011A
Adj. R^2^	0.40	0.31	0.24	0.32	0.25	0.20	0.44	0.40
AICc	261.66	272.38	244.55	236.63	−33.78	−29.34	−364.81	−359.87
Moran’s *I*	0.21	0.28	0.18	0.18	−0.17	−0.14	−0.01	0.03

^a^ Mean coefficient, ^b^ Minimum coefficient, ^c^ Maximum coefficient. A and F denote the percentage of agricultural and forested areas, respectively.

**Table 5:** Mean coefficients and confidence intervals of coefficients in estimated GWR models.

	BOD		DO		NH_3_-N		PO_4_-P	
	SWMA	Riparian	SWMA	Riparian	SWMA	Riparian	SWMA	Riparian
Mean Co. ^(1)^	−0.039F	−0.029F	−0.037A	−0.035A	−0.006F	0.005A	0.0008A	0.0007A
Confidence Interval ^(2)^	(−0.042, −0.033)	(−0.031, −0.026)	(−0.042, −0.031)	(−0.038, −0.031)	(−0.0058, −0.0056)	(0.0050, 0.0053)	(0.0007, 0.0009)	(0.0006, 0.0008)

^(1)^ Mean Coefficient. ^(2)^ 95% confidence interval (lower limit 2.5%, upper limit 97.5%). A and F denote the percentage of agricultural and forested areas, respectively. Boot resamples = 3000.

**Table 6:** Performance indicators of OLS and GWR models at both scales. The higher value of R^2^ and lower values of AICc and Moran’s *I* (absolute value) indicate better performance of the estimated models.

Water Quality	Criteria	SWMA Scale		Riparian Scale	
		OLS	GWR	OLS	GWR
BOD	Adj. R^2^	0.40	0.50	0.31	0.48
	AICc	261.66	254.24	272.38	257.79
	Moran’ *I*	0.21	0.004	0.28	0.004
DO	Adj. R^2^	0.24	0.44	0.32	0.48
	AICc	244.55	224.92	236.63	218.79
	Moran’ *I*	0.18	−0.07	0.18	−0.05
NH_3_-N	Adj. R^2^	0.25	0.25	0.20	0.24
	AICc	−33.78	−32.85	−29.34	−28.34
	Moran’ *I*	−0.17	−0.20	−0.14	−0.17
PO_4_-P	Adj. R^2^	0.44	0.49	0.40	0.44
	AICc	−364.81	−367.61	−359.87	−362.53
	Moran’ *I*	−0.01	−0.10	0.03	−0.05

**Table 7:** *t*-Test results comparing group 1 (the percentage of land use is below the mean) and group 2 (the percentage of land use is above the mean).

	Group	Mean	SD	t-Value	*p*-Value
Coefficient of % forest (BOD)	low	−0.475	0.010	−4.211	0.000 **
	high	−0.299	0.023		
Coefficient of % agriculture (DO)	low	−0.033	0.022	1.322	0.192
	high	−0.041	0.026		
Coefficient of % forest (NH_3_-N)	low	−0.006	0.0005	−5.622	0.000 **
	high	−0.005	0.0005		
Coefficient of % agriculture (PO_4_-P)	low	0.0006	0.0004	−6.792	0.000 **
	high	0.0011	0.0002		

** *p* < 0.01.
